# CCMIM: Optimizing concrete defect detection through state-space modeling and dynamic feature fusion

**DOI:** 10.1371/journal.pone.0340764

**Published:** 2026-01-27

**Authors:** Xiaozhen Li

**Affiliations:** Architectural Engineering Institute, Jinhua University of Vocational Technology, Jinhua, China; Chongqing Normal University, CHINA

## Abstract

Concrete defect detection is crucial to the safety, reliability, and durability of structures. For CNN models, it is impossible to obtain all information at different scales and complex backgrounds, nor can it capture all contexts globally. Transformer-based models are computationally intensive, making it difficult to generalize to real-time detection tasks. To address these issues, we propose a novel end-to-end concrete crack detection framework: Concrete Crack Mamba-in-Mamba (CCMIM). Specifically, we introduce the Mamba-In-Mamba (MiM) module to capture long-range dependencies and global context to improve the concrete defect detection capability based on hierarchical data flow. In addition, this paper also proposes the Dynamic Dual Fusion (DDF) module, which enhances the robustness and adaptability of the model and achieves smooth multi-scale fusion by dynamically changing the feature representation. To reduce the computational cost and maintain spatial information, we propose the Sparse Pyramid Transformer (SPT) module. This module reduces the computation and improves the inference speed by selecting tokens level by level (from coarse to fine) and sharing attention parameters, but does not sacrifice accuracy. Experimental results show that the CCMIM model outperforms traditional methods as well as YOLO- and Transformer-based models in small crack detection across multiple datasets. Specifically, on the RDD2022, SDNET2018, and CCCD datasets, the accuracy reached 89.2%, 85.2%, and 79.3%, respectively, while the mAP50 reached 88.1%, 87.8%, and 79.2%. In summary, the CCMIM model provides an effective solution for concrete defect detection. The code can be accessed at: https://github.com/lixiaozhen01/CCMIM.

## 1 Introduction

As urbanization accelerates, concrete structures have been widely applied in infrastructure such as roads, buildings, and bridges [1–3]. To ensure the long-term safety and stability of these structures, timely and accurate detection of defects in concrete (such as cracks, spalling, holes, etc.) has become increasingly important [4–6]. Traditional methods for detecting concrete defects typically rely on manual visual inspection or conventional image processing techniques. These methods are not only time-consuming but also susceptible to human factors, making it difficult to meet the demands for efficient and high-precision detection [7]. Therefore, automated and intelligent concrete defect detection methods have become a key area of research, especially in monitoring highways, bridges, and buildings, where they hold significant application potential.

In recent years, deep learning methods have made significant progress in the field of object detection [8–11]. Convolutional neural networks (CNNs), with their ability to extract local features, have become the mainstream architectural framework for early concrete defect detection. For instance, models like U-Net and its improved versions, through an encoder-decoder structure, achieve pixel-level segmentation of crack regions, demonstrating high accuracy on standard datasets in controlled laboratory environments [12]. Meanwhile, YOLO series algorithms (YOLOv8 [13], YOLOv10 [14], YOLOv11 [15]), as efficient real-time object detection algorithms, further balance detection speed and accuracy through a single-stage detection pipeline, adaptive anchor box design, and multi-scale feature fusion strategies. They are widely applied in concrete crack detection in engineering scenarios, thanks to their fast inference speed and excellent defect localization ability . Additionally, Transformer-based detection models (such as DETR [16]) break the local receptive field limitation of CNNs with self-attention mechanisms, enabling them to capture long-range feature dependencies in images. They show unique advantages in handling small cracks in complex backgrounds. For example, Deformable-DETR [17] uses deformable attention modules to focus on defect areas, reducing interference from irrelevant background features, thus further improving detection accuracy.

Despite advancements, several challenges persist in the field. Firstly, Convolutional Neural Networks (CNNs) exhibit constraints in managing variations in scale and intricate background conditions, resulting in an insufficient ability to effectively capture global contextual information [18,19]. This limitation consequently diminishes detection accuracy in scenarios characterized by complexity. Secondly, although Transformer models demonstrate proficiency in capturing long-range dependencies, their substantial computational demands present significant obstacles for implementation in real-time detection applications [20,21].

Mamba is a novel network architecture based on the State Space Model (SSM) [20]. Its design aims to overcome the limitations of traditional neural network architectures when handling long-term dependencies and complex sequential data. Although the Mamba network has achieved significant results in various tasks, there is currently no Mamba network specifically designed for concrete defect detection.

To address these shortcomings, we propose the Concrete Crack Mamba-In-Mamba (CCMIM) network. By integrating the Mamba-In-Mamba (MiM) module into the backbone network [22], our approach effectively combines both global and local information, enhancing model robustness. Specifically, we consider concrete cracks as “visual sentences" and use the external Mamba module to explore global features. Then, each visual sentence is decomposed into “visual words" and the local relations between these words are further analyzed by the internal Mamba module. Such an architecture enhances the accuracy and robustness of concrete defect detection.

**Fig 1:**
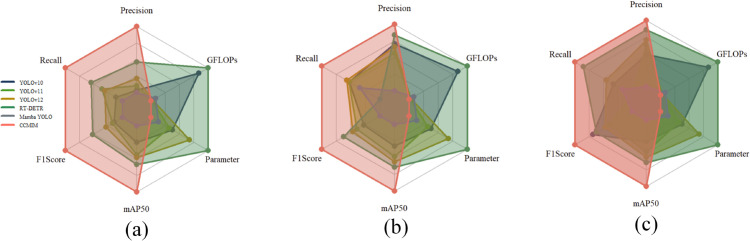
CCMIM and comparison with state-of-the-art methods. (a) Results on the RDD2022 dataset. (b) Results on the SDNET2018 dataset. (c) Results on the CCCD dataset.

In addition, Transformer-based models have achieved success in object detection, especially in handling complex scenes. Therefore, we introduce a Sparse Pyramid Transformer (SPT) module in the Neck component of the detection network. The SPT module reduces computational complexity by selecting tokens from coarse to fine and sharing attention parameters, thereby preserving spatial details. This module trains the model using coarse attention and seamlessly activates during inference, further improving detection accuracy without requiring retraining. We evaluate the proposed CCMIM model on benchmark datasets, including RDD2022, SDNET2018, and CCCD, and the experimental results demonstrate that it achieves state-of-the-art (SOTA) performance, as shown in Fig 1.

The contributions of this paper are as follows:

- We propose a novel end-to-end concrete defect detection framework. By adding the MiM module, the framework is able to capture long-range dependencies and global context information, thereby improving detection accuracy, especially in the detection of small crack features and other details.
- We innovatively introduced a dynamic dual fusion module, which dynamically adjusts feature representation to improve the robustness and adaptability of the model. It also optimizes the multi-scale feature fusion process and significantly improves the performance of the model in complex backgrounds.
- To improve computational efficiency while preserving spatial details, this paper introduces the SPT module. By selecting tokens from coarse to fine and sharing attention parameters, it reduces computational load, enhances inference speed, and ensures high-precision detection results.

The organization of this paper is as follows: Sect 2 presents related work, exploring the latest research on concrete crack detection, reviewing the current mainstream methods and their limitations, and highlighting the innovations of this paper. Sect 3 introduces the methodology, providing a detailed description of the proposed concrete defect detection framework CCMIM, including the design and implementation of the MiM block, the DDF module, and the SPT module. Sect 4 presents experiments, showcasing the results on multiple datasets and comparing them with existing methods to validate the superiority of the proposed approach. Finally, Sect 5 concludes the paper by summarizing the main contributions and discussing future directions for improvement.

## 2 Related works

### 2.1 Deep learning for concrete cracks

In concrete crack identification, traditional deep learning architectures, such as U-Net, have been widely used [10,11,23,24]. Beskopylny et al. [25] tested U-Net, LinkNet, and PSPNet models using concrete sample images obtained in laboratory tests. The results showed that the U-Net model combined with the cellular automaton algorithm performed best in terms of the following indicators: accuracy: 0.91, recall: 0.90, F1 value: 0.91, IoU: 0.84, precision: 0.90. Although the method performs well on these standard datasets, it still has limitations when dealing with complex real-world backgrounds and cracks of different sizes. In particular, U-Net performs poorly in concrete crack detection when applied to low-quality images or background noise.

In addition, in addition to deep learning-based image processing methods, Manushree N. K. Shah et al. [26] proposed an automated RCC rebar tying inspection tool to monitor RCC rebar tying in real time through image processing. However, compared with concrete crack detection, the application value of rebar tying inspection is lower, mainly focusing on verifying the quality of structural tying and ignoring surface details. Therefore, the robustness and accuracy of this method when dealing with concrete defects in more complex backgrounds are questionable and cannot meet the needs of practical applications.

Another method combines traditional imaging technology with finite element analysis for defect assessment. Miao Hong et al. [27] analyzed the temperature and deformation of defective concrete by using passive infrared thermal imaging technology and DIC method. By combining the finite element model with defect geometry and temperature analysis, the temperature field changes were deeply studied. However, this method is very sensitive to ambient temperature fluctuations and is only effective for defects that can be detected by thermal imaging. It still needs to be combined with other visual processing methods to improve its applicability.

In terms of CNN, Peile Huang et al. [28] improved the YOLOv7 architecture by adding a spatial pyramid pooling (SPP) module, thereby reducing the parameter burden and computational complexity. They also used the K-means clustering algorithm to generate anchor points and integrated the Ghost Conv module to accelerate the convergence of the model. The improved YOLOv7 model performed well on multiple datasets with good speed and accuracy. However, when faced with diverse and complex cracks, its ability to capture local features may be limited, thus affecting its performance.

The CCMIM network proposed in this paper introduces the MiM module, which combines global and local information to improve the accuracy and robustness of concrete defect detection. Compared with existing methods, the CCMIM model can better capture the diversity and details of cracks and can adjust to complex backgrounds. This provides a new and effective method for concrete defect detection.

### 2.2 Mamba network research

Mamba networks have shown great potential in multiple research areas, especially in sequence modeling, visual tasks, and medical image segmentation. Despite their excellent performance in these tasks, they still have some limitations. For example, the state-space model (SSM) [20] is good at processing long sequences and capturing long-range dependencies, but the computational cost is high when dealing with long sequences, resulting in a significant increase in training and inference time.

The bidirectional Mamba block proposed by Vim) [21] improves feature extraction performance by more effectively utilizing data. However, the complex network structure increases the computational and memory requirements, which may lead to reduced efficiency, especially on resource-constrained devices. VMamba) [29] improves the performance of visual tasks through the Mamba backbone network and cross-scan module, but at the same time increases the model complexity, especially when processing high-resolution images, the computational load and complexity increase significantly. In the field of medical image segmentation, methods such as U-Mamba) [30] and Mamba-Unet) [31] use the Mamba module to improve segmentation accuracy and effectively preserve detail information. However, the performance of these methods is still challenging when facing high-resolution images, which limits their application in large-scale datasets. P-Mamba) [32] enhances the ability to reduce background noise and preserve edge details through PM diffusion and Mamba modules, but the model is prone to errors in complex backgrounds or with multiple objects, which affects segmentation accuracy. The study of Swin-U-Mamba) [33] emphasized the importance of ImageNet pre-training for Mamba networks in medical image segmentation tasks. Although pre-training can improve performance, the transferability of pre-trained networks still needs to be further improved, especially when processing diverse medical images.

In general, although the Mamba network performs well in multiple fields, especially in visual and medical image processing tasks, its computational efficiency, memory consumption, and generalization ability still exist. In order to maximize the potential of the network, these aspects need to be further optimized and adjusted.

## 3 Methods

### 3.1 Preliminaries

State Space Models (SSM) [30] are commonly used as linear time-invariant systems. They transform the one-dimensional input signal $x(t)\in {\mathbb{R}}^{L}$ into an intermediate hidden state $h(t)\in {\mathbb{R}}^{N}$, ultimately producing the output $y(t)\in {\mathbb{R}}^{L}$. Mathematically, SSMs are often described by linear ordinary differential equations (ODEs), where the system is characterized by a set of parameters including the state transition matrix $A\in {\mathbb{C}}^{N\times N}$, the projection matrix $B\in {\mathbb{C}}^{N}$, and the jump connection matrix $D\in {\mathbb{C}}^{1}$, as shown in Eq 1:

$$h^{\prime }(t)=Ah(t)+Bx(t)\;y(t)=Ch(t)+Dx(t)$$ (1)

When applying State Space Models (SSM) to deep learning tasks, challenges arise due to their continuous time characteristics. To address this issue, the ordinary differential equation (ODE) needs to be converted into a discrete function. Suppose the input is $x_{k}\in {\mathbb{R}}^{L\times D}$, which represents the sampled vector with length *L* from the signal flow. As described in reference [45], to discretize the continuous parameters *A* and *B*, we introduce the time-domain parameter Δ, and obtain their discrete versions $\bar{A}$ and $\bar{B}$, following the zero-order hold (ZOH) rule. Thus, Eq 1 is discretized as follows:

$$h_{k}=\bar{A}h_{k-1}+\bar{B}x_{k}\;y(t)=Ch(t)+Dx(t)\;\bar{A}=e^{\Delta A},\;\bar{B}=(e^{\Delta A}-I)A^{-1}B,\;\bar{C}=C$$ (2)

Where, $\bar{A}$ and $\bar{B}$ are the discretized parameters, and $B,C\in {\mathbb{R}}^{D}$, $\Delta \in {\mathbb{R}}^{D}$, representing the sampling time interval vector during the discretization of continuous-time signals. In practical applications, the discretization process is further optimized by approximating $\bar{B}$ using a first-order Taylor expansion:

$$\bar{B}=(e^{\Delta A}-I)A^{-1}B=\Delta A(\Delta A{)}^{-1}AB=\Delta B$$ (3)

### 3.2 Propose network

This paper proposes a new end-to-end concrete defect detection framework, the CCMIM network. The network integrates a Mamba-based backbone network, a dynamic dual fusion (DDF) module, and an SPT module. The goal is to improve the accuracy and efficiency of concrete defect detection by efficiently fusing global and local information. The Mamba-In-Mamba (MiM) module is used to capture long-range dependencies and global context information, and the DDF module dynamically adjusts the feature representation to enhance the robustness and adaptability of the model. In order to optimize the computational complexity and preserve spatial details, the network introduces the SPT module, which reduces the computational burden by selecting tokens from coarse to fine and sharing attention parameters.

**Fig 2:**
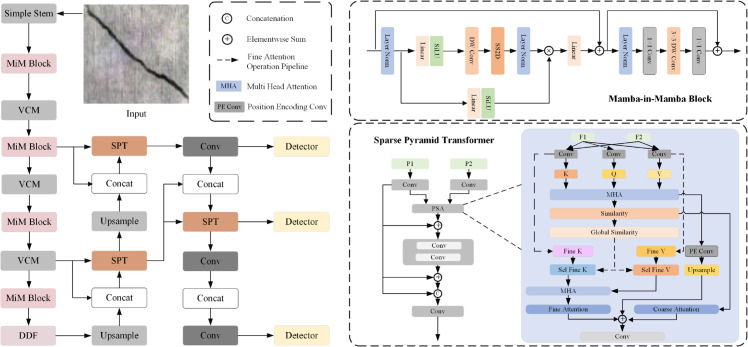
CCMIM network architecture diagram. The model captures and enhances local features through multiple MiM modules, while the SPT module is used for multi-scale feature fusion, improving the model’s computational efficiency. The DDF module strengthens the fusion of fine-grained and coarse-grained features, enhancing feature discriminability.

As shown in the Fig 2, the architecture of the CCMIM network starts with a simple Stem module, which is responsible for extracting initial features. The MiM block module gradually enhances the feature representation. The VCM module further refines the extracted features to ensure the completeness and accuracy of the details. The DDF module replaces the traditional SPPF module and consists of two complementary components: ECA (enhanced channel attention) and dynamic filter (DF). ECA extracts and aggregates contextual features through the global average pooling (GAP) mechanism, and DF dynamically adjusts the importance of features according to the input to enhance the flexibility and accuracy of the model. In the neck part of the network, the SPT module reduces the computational complexity and retains spatial details through a sophisticated attention mechanism. Finally, the convolution operation and detection module output the final detection result.

### 3.3 Mamba-in-Mamba

Concrete defect detection, as an important application in the field of computer vision, faces several challenges. Traditional defect detection methods often rely on Convolutional Neural Networks (CNN) or a combination of CNNs and Vision Transformers (ViT). While these methods have achieved significant results in object detection and image classification tasks, their performance still faces limitations when dealing with small objects, capturing detailed features, and handling complex backgrounds. Specifically, for detecting fine defects like concrete cracks, precise local feature extraction is key to improving detection accuracy.

The Mamba-in-Mamba (MiM-ISTD) model [22], proposed by Tianxiang Che et al., provides an important reference for the collaborative extraction of global and local features in concrete defect detection. By employing hierarchical feature modeling and an efficient computational architecture, this model achieves the cooperative extraction of local and global features while maintaining linear computational complexity. It is well-suited for high-resolution infrared image scenes and has shown excellent capabilities in capturing fine details and computational efficiency in small target infrared detection tasks. The core idea lies in using the nested Mamba structure to handle features at different scales, offering an effective solution to the conflict between “global context awareness" and “local detail retention."

To meet the needs of concrete defect detection, this paper introduces the Mamba-in-Mamba (MiM) Block as a core component of the backbone network. The MiM Block employs a “visual sentence - visual word" hierarchical feature parsing mechanism to precisely capture both the global distribution of concrete cracks and local texture features. The specific process is as follows:

The input feature map $F_{\text{in}}\in {\mathbb{R}}^{C_{\text{in}}\times H\times W}$ (where $C_{\text{in}}$ is the number of input channels, and *H* and *W* are the height and width of the feature map) is first processed with Layer Normalization to eliminate the impact of feature distribution differences across channels, ensuring stability in deep network propagation. The equation is as follows:

$$F_{\text{ln}}=\text{LayerNorm}(F_{\text{in}})=\gamma \cdot \frac{F_{\text{in}}-\mu }{\sqrt{\sigma^{2}+\epsilon }}+\beta$$ (4)

where μ and $\sigma^{2}$ are the mean and variance across the channel dimension of the input feature map, γ and β are learnable scale and shift parameters, and ϵ is a small value (typically 10^−5^) to avoid division by zero.

The normalized feature $F_{\text{ln}}$ is then passed through a linear layer to map and transform the features into a dimension suitable for the Mamba module, producing an initial feature representation $F_{\text{lin}}$. The equation is as follows:

$$F_{\text{lin}}=W_{\text{lin}}\cdot F_{\text{ln}}+b_{\text{lin}}$$ (5)

where $W_{\text{lin}}\in {\mathbb{R}}^{C_{\text{mid}}\times C_{\text{in}}}$ and $b_{\text{lin}}\in {\mathbb{R}}^{C_{\text{mid}}}$ are the weight matrix and bias vector of the linear layer, and $C_{\text{mid}}$ is the number of intermediate feature channels.

To enhance the nonlinear representation of features, we apply the SiLU activation function to $F_{\text{lin}}$, highlighting the feature differences between concrete cracks and the background. The equation is as follows:

$$F_{\text{silu}}=\text{SiLU}(F_{\text{lin}})=F_{\text{lin}}\cdot \sigma (F_{\text{lin}})$$ (6)

where σ(·) represents the Sigmoid function. This activation function adaptively adjusts the feature response strength, effectively amplifying the signal from the crack regions.

A Depthwise Convolution (DW Conv) module is applied to $F_{\text{silu}}$ for local feature extraction. Unlike traditional convolutions, Depthwise Convolution applies convolution kernels independently to each feature channel, reducing computational complexity while allowing for finer extraction of local crack features, such as crack edges and width variations. The equation is as follows:

$$F_{\text{dw}}[c,i,j]=\sum_{k=0}^{K-1}\sum_{l=0}^{K-1}W_{\text{dw}}[c,k,l]\cdot F_{\text{silu}}[c,i+k,j+l]+b_{\text{dw}}[c]$$ (7)

where $W_{\text{dw}}\in {\mathbb{R}}^{C_{\text{mid}}\times K\times K}$ is the depthwise convolution kernel (with kernel size *K* = 3), and $b_{\text{dw}}\in {\mathbb{R}}^{C_{\text{mid}}}$ is the bias vector for Depthwise Convolution.

To achieve the collaborative use of global and local features, the concrete crack features are parsed hierarchically as “visual sentence - visual word":

- Visual Sentence Division: The feature map $F_{\text{dw}}$ is treated as a set of “visual sentences" by performing a spatial partitioning operation, dividing $F_{\text{dw}}$ into *N* non-overlapping spatial blocks $S_{1},S_{2},...,S_{N}$ (each block size is s×s, such as 4×4), each corresponding to a “visual sentence" $S_{n}\in {\mathbb{R}}^{C_{\text{mid}}\times s\times s}$. The equation is:
  $$S_{n}=F_{\text{dw}}[:,n_{s}\cdot s:(n_{s}+1)\cdot s,n_{s}\cdot s:(n_{s}+1)\cdot s]\;(n=1,2,...,N)$$ (8)
  where *n*_*s*_ is the index of the spatial block.
- Visual Word Decomposition: Each “visual sentence" *S*_*n*_ is further decomposed into “visual words" by grouping the channel dimension into *M* groups $G_{1},G_{2},...,G_{M}$, where each group corresponds to a “visual word" $W_{n,m}\in {\mathbb{R}}^{(C_{\text{mid}}/M)\times s\times s}$. The equation is:
  $$W_{n,m}=S_{n}[(m-1)\cdot (C_{\text{mid}}/M):m\cdot (C_{\text{mid}}/M),:,:]$$ (9)
  This step refines the channel features of each “visual sentence" into multiple local feature groups, ensuring the precise capture of fine details of the cracks (e.g., pixel-level texture, small fractures).

Finally, the SS2D (State Space 2D) module is used to fuse the global dependencies of the “visual sentences" with the local features of the “visual words". The SS2D module is based on a State Space Model (SSM) that models the global features of each “visual sentence" and enhances the correlation of the local features of the “visual words". The fused feature map $F_{\text{ss2d}}$ is produced by the equation:

$$F_{\text{ss2d}}=SS2D(\{S_{n}\},\{W_{n,m}\})=\sum_{n=1}^{N}\sum_{m=1}^{M}\alpha_{n,m}\cdot SSM(W_{n,m})+\beta_{n}\cdot S_{n}$$ (10)

where $\alpha_{n,m}$ and $\beta_{n}$ are learnable attention weights, and SSM(·) is the feature transformation function of the State Space Model used to capture long-range dependencies.

After the SS2D module, the feature map undergoes a linear transformation to adjust the dimensionality for subsequent calculations. Then, a second Layer Normalization is applied to further enhance the stability and consistency of the features. Following this, the feature map is processed with the GELU activation function, along with 1x1 convolution and 3x3 depthwise convolution, capturing more complex spatial information. The final output feature is $F_{\text{mim}}$. Through this hierarchical feature parsing and fusion method, the MiM module can precisely capture both global and local features of concrete cracks, greatly enhancing defect detection accuracy and efficiency.

### 3.4 Vision clue merge

**Fig 3:**
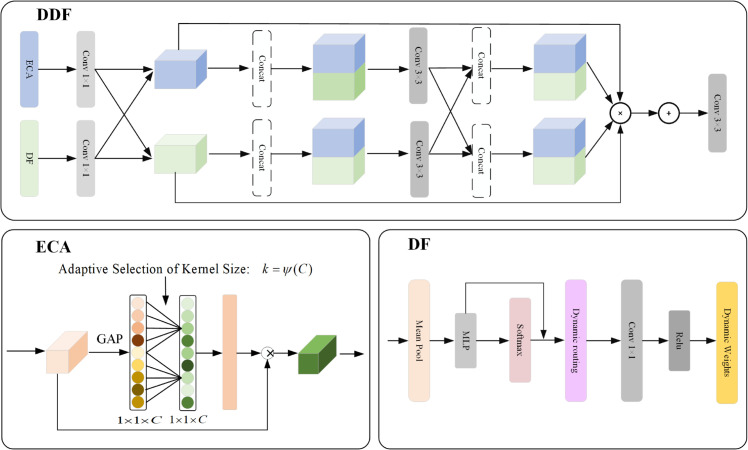
Illustration of the vision clue merge block.

To preserve informative visual cues during downsampling, we utilized the Vision Clue Merge (VCM) Block introduced in VMamba [29]. As shown in Fig 3, we first spatially split the feature map and then compress it using 1×1 pointwise convolutions. These compressed features are concatenated along the channel axis and further reduced by another pointwise convolution, achieving an overall fourfold spatial compression.

### 3.5 Dynamic dual fusion module

**Fig 4:**
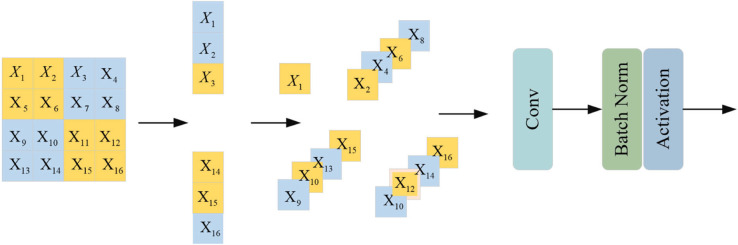
DDF network architecture diagram. It integrates ECA and DF mechanisms to improve feature fusion and discriminability.

In order to effectively fuse multi-scale contextual features and enhance the distinguishability of features, this paper proposes integrating the Dynamic Dual Fusion (DDF) module into the network architecture (as shown in Fig 4). The module innovatively combines the Enhanced Channel Attention (ECA) and Dynamic Filter (DF) mechanisms, using differentiated convolution operations and feature channel fusion strategies, allowing the model to adaptively match the convolution kernel size and dynamic weights, significantly improving feature expressiveness and providing better feature support for concrete crack detection, especially for small cracks in complex backgrounds.

The ECA module focuses on “channel-level attention optimization." It aggregates the channel dimension information of the feature map $F_{\text{mim}}\in {\mathbb{R}}^{C_{\text{in}}\times H\times W}$ (where $C_{\text{in}}$ is the input channel number, and *H* and *W* are the height and width of the feature map) through global average pooling (GAP) to generate channel-level global feature statistics. Its uniqueness lies in adaptively selecting the convolution kernel size: in concrete crack detection, where “crack features are scattered across different channels (such as edge channels and texture channels)," ECA models the inter-channel dependencies directly without dimensionality reduction. By dynamically adjusting the kernel size (e.g., choosing a 3×3 or 5×5 kernel based on the crack scale), it precisely captures key channel features related to the crack while suppressing redundant channel responses from the background noise. This design avoids information loss due to dimensionality reduction in traditional channel attention (such as SE modules) and enhances the channel weight of crack features, allowing the model to focus on the effective features in the crack region, even under complex backgrounds (e.g., surface stains or texture interference in concrete).

The DF module focuses on “spatial feature dynamic adjustment" by using a multi-layer perceptron (MLP) and dynamic routing mechanisms to adaptively optimize the spatial features of $F_{\text{mim}}$. Its uniqueness lies in the dynamic generation and spatial adaptation of filter parameters: for concrete cracks, which have “irregular shapes (such as linear cracks, network cracks) and significant scale differences (from millimeter-level fine cracks to centimeter-level wide cracks)," the DF module does not rely on fixed convolution kernels. Instead, it dynamically generates adapted filter parameters based on the local spatial features of $F_{\text{mim}}$ (such as crack edge gradients and pixel intensity variations)—high-resolution filters are generated in crack edge areas to retain fine details, and low-resolution filters are generated in smooth crack areas to reduce computational redundancy. Meanwhile, the dynamic routing mechanism adjusts the spatial coverage of the filters in real-time to ensure accurate feature extraction for different crack shapes, avoiding feature extraction biases seen in traditional fixed convolution kernels in highly variable crack morphology scenarios.

Existing dynamic convolution networks (e.g., DyConv, CondConv) have achieved dynamic kernel adjustment, but they focus primarily on “single-dimension optimization" (such as dynamically adjusting convolution kernel parameters or optimizing channel attention) and often use “static weighting" fusion mechanisms, which are difficult to adapt to the multi-scale, multi-morphology, and complex background requirements of concrete crack detection. The dynamic fusion mechanism proposed in this paper is significantly innovative in the following aspects:

The dynamic fusion mechanism proposed in this paper (used for the DDF module) represents a significant innovation compared to existing dynamic convolution networks such as DyConv and CondConv: existing networks focus primarily on single-dimension optimization (e.g., adjusting convolution kernel parameters or optimizing channel attention) and use static weighted fusion, making them difficult to adapt to the multi-scale, multi-morphology, and complex background requirements of concrete crack detection. In contrast, the proposed mechanism builds a “channel - spatial dual-dimension dynamic adjustment system" through the collaboration of ECA (channel-level optimization) and DF (spatial-level optimization). This resolves the issues of existing networks losing key channel information or neglecting spatial details. Based on the real-time feature statistics of $F_{\text{mim}}$, the adaptive weight generation algorithm dynamically allocates the fusion ratio of ECA and DF output features, replacing traditional static weighting to adapt to real-time changes in crack features. Furthermore, the ECA module achieves dimensionality reduction-free modeling, DF parameter sharing, and simplified branch design to achieve lightweight performance, balancing both performance and efficiency, making it more suitable for concrete defect detection requirements.

### 3.6 Sparse pyramid transformer

In this paper, we use the SPT network for multi-scale fusion, and we are able to capture global context and local information at the same time. The combination of these techniques makes the detection process more accurate and robust, and enhances the model’s ability to detect details on complex concrete surfaces.

The network input includes two feature maps *F*_1_ and *F*_2_, where $F_{1}\in {\mathbb{R}}^{C\times H\times W}$ is a high-resolution feature map, and $F_{2}\in {\mathbb{R}}^{C^{\prime }\times \frac{H}{2}\times \frac{W}{2}}$ is a low-resolution feature map. These feature maps are convolved to obtain $F_{1}^{\prime }$ and $F_{2}^{\prime }$.

$$F_{1}^{\prime }=\text{Conv}(F_{1},K_{1}),\;F_{2}^{\prime }=\text{Conv}(F_{2},K_{2})$$ (11)

where *K*_1_ and *K*_2_ are convolution kernels. After the convolution operation, the dimensions of the feature maps are altered, setting the foundation for subsequent multi-scale feature extraction and fusion.

After convolution, the feature maps $F_{1}^{\prime }$ and $F_{2}^{\prime }$ enter the pyramid sparse attention (PSA) module (As shown in Algorithm 1). PSA enhances feature representation through multi-scale convolution and self-attention mechanism to capture information of different receptive fields. The PSA module outputs ${F_{1}^{\prime }}^{\prime }$ and ${F_{2}^{\prime }}^{\prime }$:

$$F_{1}^{\prime \prime }=\text{PSA}(F_{1}^{\prime }),\;F_{2}^{\prime \prime }=\text{PSA}(F_{2}^{\prime })$$ (12)

The PSA module enhances the representation capability of feature maps through spatial and channel attention mechanisms, providing higher quality feature input for subsequent multi-head attention and feature fusion.

After being processed by the PSA module, the feature maps $F_{1}^{\prime \prime }$ and $F_{2}^{\prime \prime }$ are extracted through the multi-head self-attention (MHA) mechanism, which captures the relationship between each position in the feature map, especially the long-range dependency. The query (Q), key (K), and value (V) are generated through the convolution operation:

$$Q=\text{Conv}(F_{1}^{\prime \prime }),\;K=\text{Conv}(F_{2}^{\prime \prime }),\;V=\text{Conv}(F_{1}^{\prime \prime })$$ (13)

The similarity between the queries and keys is computed and normalized:

$$\text{Attention}(Q,K,V)=\text{softmax}\left(\frac{QK^{T}}{\sqrt{d_{k}}}\right)V$$ (14)

This process adjusts the importance of different features by weighting the value (V), allowing the network to focus more on identifying small objects.

The network uses fine-grained attention and coarse-grained attention modules to process fine-grained and coarse-grained features. The fine-grained attention module processes small object details, and the coarse-grained attention module helps capture global information. This stage processes fine-grained and coarse-grained features separately and optimizes their representation capabilities:

$$F_{\text{fine}}=\text{FineAttention}(F_{1}^{\prime \prime },F_{2}^{\prime \prime }),\;F_{\text{coarse}}=\text{CoarseAttention}(F_{1}^{\prime \prime },F_{2}^{\prime \prime })$$ (15)

This design enables the network to extract local details and global context respectively when dealing with complex backgrounds and tiny cracks, thereby enhancing its ability to handle features of different scales.

Through the concatenation operation, the outputs of the fine-grained attention and coarse-grained attention modules $F_{\text{fine}}$ and $F_{\text{coarse}}$ are combined into one feature map:

$$F_{\text{concat}}=\text{Concat}(F_{\text{fine}},F_{\text{coarse}})$$ (16)

Then, the fused feature map $F_{\text{concat}}$ undergoes the convolution operation for the final feature fusion, and the enhanced feature map $F_{\text{final}}$ is obtained:

$$F_{\text{final}}=\text{Conv}(F_{\text{concat}},K_{3})$$ (17)

Finally, the fused feature map is processed by the upsample module to upsample the feature map to the original input size for subsequent processing or output. The upsampled feature map $F_{\text{output}}$ represents the final output of the network:

$$F_{\text{output}}=\text{Upsample}(F_{\text{final}})$$ (18)

In summary, by combining the MiM module, SPT module and DDF module, the framework effectively extracts multi-scale features and enhances the fusion of local details and global context. Experimental results show that the network has significant advantages in processing complex backgrounds and detecting small cracks, improving the accuracy and robustness of defect detection.

**Algorithm 1. Sparse pyramid transformer model.**



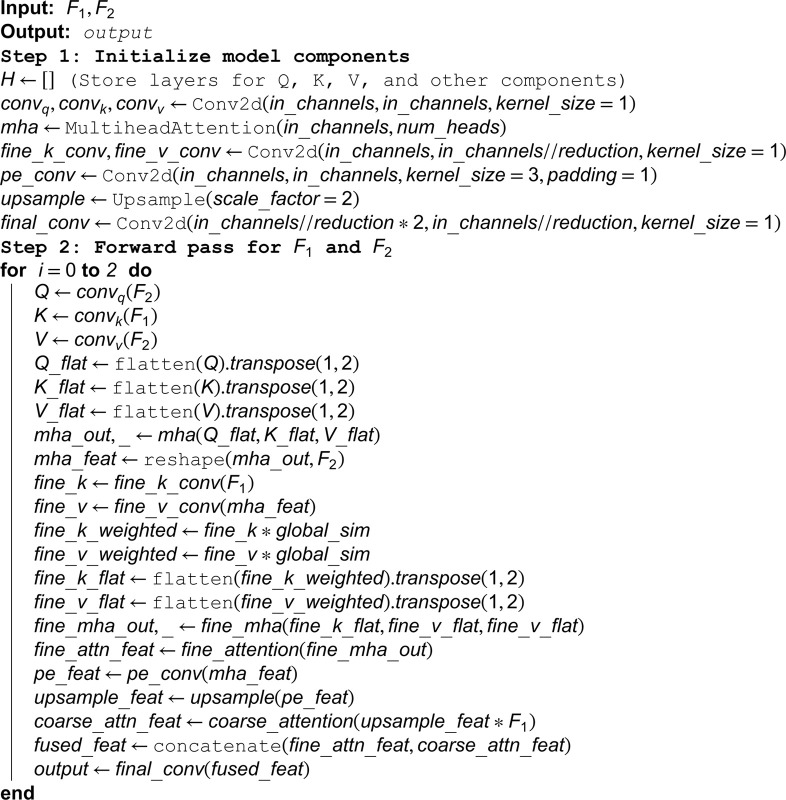



## 4 Experiment

### 4.1 Experimental setup

**Datasets.** This paper uses three datasets that are widely used in defect detection tasks, namely RDD2022 [34], SDNET2018 [35], and CCCD [36] datasets. The RDD2022 dataset is used for road defect detection. It contains 23,767 images covering various road damage types such as cracks, potholes, and wear, and is accurately annotated to provide high-quality training and test data. The SDNET2018 dataset contains 56,000 images of concrete bridge decks, covering both cracked and non-cracked conditions. It is suitable for concrete bridge deck crack detection tasks and promotes the intelligent and automated research of bridge maintenance. The CCCD dataset focuses on concrete crack detection and contains about 3,500 images, covering crack images of different types and environmental conditions. It provides rich samples and annotations for concrete defect detection and is suitable for the training and evaluation of deep learning models.

**Experimental Environment.** Table 1 presents the experimental environment used in this study. The experiments were conducted on a high-performance computing platform equipped with modern GPUs, enabling efficient parallel processing of deep learning tasks.

**Table 1:** Experimental environment configuration.

Category	Configuration
Hardware	CPU: Intel Core i9-10900K (10 cores, 3.7 GHz)
	GPU: NVIDIA RTX 3090 (24 GB VRAM)
	RAM: 64 GB DDR4
	Storage: 1 TB SSD
Software	Operating System: Ubuntu 20.04 LTS
	Deep Learning Framework: PyTorch 1.10.0
	Python: 3.8
	CUDA: 11.3
	Libraries: NumPy 1.21.0, SciPy 1.7.0, OpenCV 4.5.3

**Hyperparameter.** As shown in Table 2, the hyperparameter settings for the study are presented, including key parameters such as learning rate, batch size, and training epochs. Additionally, this study employs a step decay strategy to dynamically adjust the learning rate. The specific settings are as follows: the initial learning rate is set to 0.01, and the learning rate is reduced by a factor of 0.1 every 50 epochs (i.e., 1/3 of the total 150 training epochs). After the 50th epoch, the learning rate is reduced to 0.001, and after the 100th epoch, it is further reduced to 0.0001. This setup allows the model to converge quickly in the early stages of training with a higher learning rate, efficiently learning the basic features of concrete cracks, while fine-tuning parameters with a lower learning rate in the later stages to precisely capture fine-grained features of small cracks, avoiding parameter oscillations.

**Table 2:** Hyperparameter configuration.

Hyperparameter	Configuration
Learning Rate	0.01
Image Size	640*640
Momentum	0.937
Optimizer	SGD
Batch Size	32
Epoch	150
Weight Decay	0.0005
close_mosaic	10
Learning rate	0.01

### 4.2 Evaluation metrics

In this paper, we use some metrics to evaluate the performance of the model: precision (P), recall (R), F1 score, and mean average precision at 50 (mAP50). P measures the proportion of true +ve samples in all the +ve sample samples predicted as positive by the model, while Recall (R) evaluates the proportion of True positive samples correctly identified by the model amongst all actual positive samples. F1 score is the Harmonic mean of Precision and Recall, giving an indication on the balance between them Of these two, it is especially important for imbalanced class problems. Similarly, mAP50 calculates average precisions at IoU=50% for each class, refers to the effectiveness of the model in all detection tasks. These metrics collectively Help us form a fuller evaluation of the model’s efficiency and precision in concrete defect detection.

### 4.3 Quantitative comparison results

#### RDD2022 dataset detection results.

As shown in Table 3, CCMIM achieved the best performance on the RDD2022 dataset, with a precision of 89.2%, recall of 82.6%, F1 score of 85.8%, and mAP50 of 88.1%. Compared to representative YOLO models, such as YOLOv12, CCMIM improved precision by 4.4 percentage points, recall by 5.4 percentage points, F1 score by 4.9 percentage points, and mAP50 by 3.9 percentage points. Compared to Transformer-based models (such as Deformable-DETR), CCMIM improved precision by 6.1 percentage points, recall by 7.2 percentage points, F1 score by 6.6 percentage points, and mAP50 by 6.6 percentage points. Even when compared to RT-DETR, a Transformer model with higher precision, CCMIM improved precision by 3.0 percentage points, recall by 3.5 percentage points, F1 score by 3.3 percentage points, and mAP50 by 3.1 percentage points.

**Table 3:** Results comparison of different algorithms with the RDD2022 dataset. The best results are displayed in bold.

Model	P	R	F1 Score	mAP50	Para(M)	GFLOPs	FPS
Faster R-CNN [37]	68.9	59.3	63.9	63.5	68.47	122.4	67
CenterNet [38]	71.8	62.4	66.9	69.1	14.4	19.4	263
RADNet [39]	86.6	78.7	82.5	83.5	**2.47**	9.4	370
YOLOv3 [40]	82.9	76.6	79.5	83.1	103.69	282.7	106
YOLOv5s [41]	77.6	69.6	73.4	75.9	2.61	7.9	303
YOLOv6n [42]	76.3	65.4	70.5	71.7	4.23	11.8	303
YOLOv7 [43]	71.3	55.4	57.8	57.9	4.55	13.1	278
YOLOv8n [44]	78.3	71.4	78.4	78.2	3.02	8.1	435
YOLOv9 [45]	82.9	74.6	78.6	80.5	25.32	12.3	175
YOLOv10 [14]	83.8	75.7	79.9	82.5	15.31	35.0	215
YOLOv11 [15]	84.2	77.6	80.1	83.9	13.58	6.8	279
YOLOv12 [46]	84.8	77.2	80.9	84.2	25.32	7.3	272
Deformable-DETR [17]	83.1	75.4	79.2	81.5	42.3	36.8	35
Conditional-DETR-DC5 [47]	84.0	77.3	80.6	82.5	48.7	52.2	40
Efficient-DETR [48]	85.2	78.0	81.5	84.0	34.5	28.9	45
SMCA-DETR [49]	85.4	78.2	81.8	84.2	50.1	47.3	38
RT-DETR-R50 [50]	86.2	79.1	82.5	85.0	36.2	40.5	50
MiM-ISTD [22]	82.5	74.3	77.7	80.1	6.2	7.3	412
Voxel Mamba [51]	84.1	75.5	79.7	81.9	6.8	8.2	437
Mamba YOLO [52]	83.6	74.8	78.9	80.6	7.2	9.0	435
**CCMIM**	**89.2**	**82.6**	**85.8**	**88.1**	2.88	**6.3**	**450**

In terms of computational efficiency, CCMIM also shows significant advantages: it has only 2.88M parameters and 6.3 GFLOPs of computation, which is much lower than Deformable-DETR and RT-DETR. Additionally, it achieves a frame rate of 450 FPS, which is higher than YOLOv12 (272 FPS), striking a balance between detection accuracy and real-time performance, making it suitable for efficient detection of road concrete defects.

#### SDNET2018 dataset detection results.

As shown in Table 4, we conducted bridge crack detection experiments on the SDNET2018 dataset. The results demonstrate that the CCMIM (Concrete Crack Mamba-in-Mamba) model outperforms other comparison methods across several key metrics. Compared to YOLO series models (taking YOLOv12 as an example, with precision of 82.7 and recall of 74.3), CCMIM improved precision by approximately 2.5 percentage points, recall by about 5.2 percentage points, F1 score by around 4.6 percentage points, and mAP50 by approximately 7.8 percentage points. This indicates that CCMIM performs better in capturing subtle details of bridge concrete cracks and handling complex backgrounds, showing a significant advantage in the accurate detection of bridge concrete cracks. Compared to the best-performing RT-DETR-R50, CCMIM improved precision by about 1.2 percentage points, recall by approximately 2.5 percentage points, F1 score by around 2.1 percentage points, and mAP50 by about 5.3 percentage points, fully demonstrating CCMIM’s stronger ability to recognize small targets and fine cracks, as well as its superior detection robustness in complex scenes.

**Table 4:** Results comparison of different algorithms with the SDNET2018 dataset. The best results are displayed in bold.

Model	P	R	F1 Score	mAP50	Para(M)	GFLOPs	FPS
Faster R-CNN	65.2	58.0	61.3	63.0	68.47	122.4	60
CenterNet	66.9	59.1	62.7	67.5	14.4	19.4	230
RADNet	82.0	74.5	77.9	79.0	**2.47**	9.4	310
YOLOv3	78.6	71.2	74.8	77.0	103.69	282.7	90
YOLOv5s	73.2	65.3	69.1	72.5	2.61	7.9	260
YOLOv6n	72.1	63.4	67.4	70.0	4.23	11.8	280
YOLOv7	67.2	52.1	56.2	55.5	4.55	13.1	250
YOLOv8n	75.4	68.0	71.6	73.0	3.02	8.1	400
YOLOv9	79.3	72.0	75.4	77.5	25.32	12.3	150
YOLOv10	80.1	72.8	76.2	78.5	15.31	35.0	185
YOLOv11	81.0	73.5	76.5	79.0	13.58	6.8	230
YOLOv12	82.7	74.3	77.7	80.0	25.32	7.3	240
Deformable-DETR	80.5	73.2	76.5	75.0	42.3	36.8	28
Conditional-DETR-DC5	80.6	72.8	76.6	78.0	48.7	52.2	33
Efficient-DETR	83.0	75.5	79.0	81.0	34.5	28.9	38
SMCA-DETR	83.2	76.0	79.4	81.0	50.1	47.3	30
RT-DETR-R50	84.0	77.0	80.2	82.5	36.2	40.5	40
MiM-ISTD	79.0	72.0	75.4	77.0	6.2	7.3	350
Voxel Mamba	80.3	73.4	76.7	79.5	6.8	8.2	410
Mamba YOLO	79.0	71.2	74.5	76.5	7.2	9.0	380
**CCMIM**	**85.2**	**79.5**	**82.3**	**87.8**	2.88	**6.3**	**400**

Additionally, in terms of computational complexity, CCMIM also shows a clear advantage: its parameter count is only 2.88M, much lower than RT-DETR-R50’s 36.2M, and lower than YOLOv12’s 25.32M. The computational load is only 6.3 GFLOPs, significantly lower than RT-DETR-R50’s 40.5 GFLOPs and also lower than YOLOv12’s 7.3 GFLOPs. In terms of frame rate, CCMIM reaches 400 FPS, which is higher than RT-DETR-R50’s 40 FPS and YOLOv12’s 240 FPS. This ensures high detection accuracy while effectively reducing computational costs and improving inference speed, making it better suited for practical engineering applications in bridge crack detection.

**CCCD dataset detection results.** As shown in Table 5, we conducted experiments on building crack detection using the CCCD dataset. The results indicate that the CCMIM method significantly outperforms other comparison methods across multiple key performance metrics. Specifically, in terms of precision (P), recall (R), F1-score, and mAP50, CCMIM achieved 79.3, 77.2, 78.2, and 79.2, respectively. Compared to RT-DETR-R50, CCMIM improved precision by approximately 1.1 percentage points, recall by about 6.2 percentage points, F1-score by around 3.7 percentage points, and mAP50 by approximately 3.2 percentage points. This improvement fully demonstrates that CCMIM is more effective in capturing fine cracks and more robust against interference in complex backgrounds, helping to reduce both missed detections and false positives in building crack detection.

**Table 5:** Results comparison of different algorithms with the CCCD dataset. The best results are displayed in bold.

Model	P	R	F1 Score	mAP50	Para(M)	GFLOPs	FPS
Faster R-CNN	60.5	54.1	57.0	60.2	68.47	122.4	50
CenterNet	63.2	56.1	59.5	62.0	14.4	19.4	200
RADNet	77.0	68.0	72.0	74.5	**2.47**	9.4	290
YOLOv3	70.2	63.5	66.8	70.1	103.69	282.7	80
YOLOv5s	67.5	60.1	63.5	67.0	2.61	7.9	220
YOLOv6n	66.1	58.5	61.5	64.0	4.23	11.8	250
YOLOv7	62.5	48.9	54.6	53.5	4.55	13.1	230
YOLOv8n	71.2	63.8	67.2	69.8	3.02	8.1	360
YOLOv9	74.6	66.8	70.5	72.0	25.32	12.3	140
YOLOv10	75.3	67.1	71.1	73.2	15.31	35.0	160
YOLOv11	76.4	68.2	72.3	74.6	13.58	6.8	210
YOLOv12	77.0	69.0	73.0	75.3	25.32	7.3	220
Deformable-DETR	75.6	68.3	71.4	73.0	42.3	36.8	25
Conditional-DETR-DC5	75.3	68.0	71.5	73.2	48.7	52.2	28
Efficient-DETR	77.2	69.6	73.1	74.5	34.5	28.9	32
SMCA-DETR	77.4	69.9	73.6	74.9	50.1	47.3	28
RT-DETR-R50	78.2	71.0	74.5	76.0	36.2	40.5	35
MiM-ISTD	72.1	66.2	69.0	71.5	6.2	7.3	300
Voxel Mamba	73.4	67.0	70.1	72.8	6.8	8.2	370
Mamba YOLO	72.5	64.7	68.4	70.3	7.2	9.0	340
**CCMIM**	**79.3**	**77.2**	**78.2**	**79.2**	2.88	**6.3**	**390**

The CCMIM method has shown excellent performance in road, bridge and building crack detection tasks. Through multi-scale feature fusion, powerful local feature extraction capabilities and adaptive feature fusion mechanism, CCMIM can effectively cope with small crack detection in different scenarios, overcoming the shortcomings of traditional methods and other modern methods in detail capture and complex background processing. Especially in complex environments such as road cracks, bridge concrete cracks, and building cracks, CCMIM can accurately identify tiny cracks and effectively reduce missed detections and false detections. Compared with the existing YOLO series and Transformer methods, CCMIM has significantly improved detection accuracy, recall rate, and computational efficiency. It can simultaneously ensure high accuracy and real-time reasoning capabilities, and is suitable for large-scale actual detection tasks. Through the combination of MiM module, PST module, and DDF module, CCMIM not only improves the detection accuracy of the model, but also enhances the model’s adaptability to complex scenes and tiny cracks, making it perform well in a variety of application scenarios.

### 4.4 Qualitative analysis results

**Fig 5:**
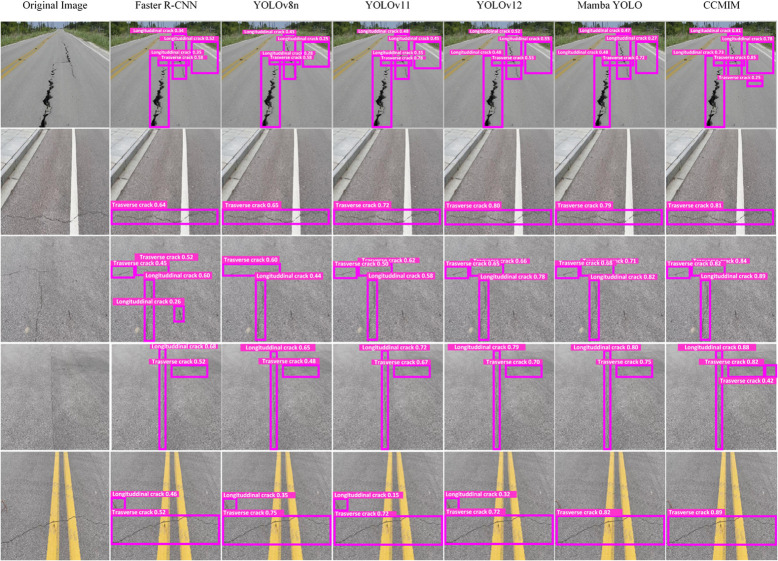
Qualitative comparison results of CCMIM on the RDD2022 dataset.

**Fig 6:**
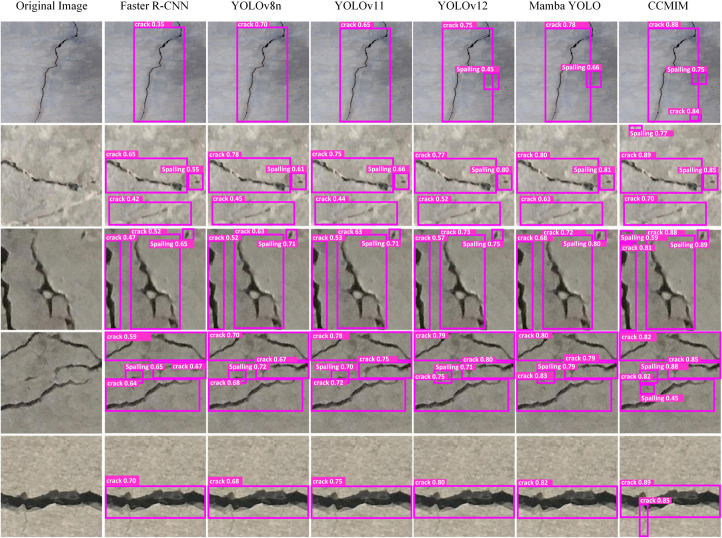
Qualitative comparison results of CCMIM on the SDNET2018 dataset.

**Fig 7:**
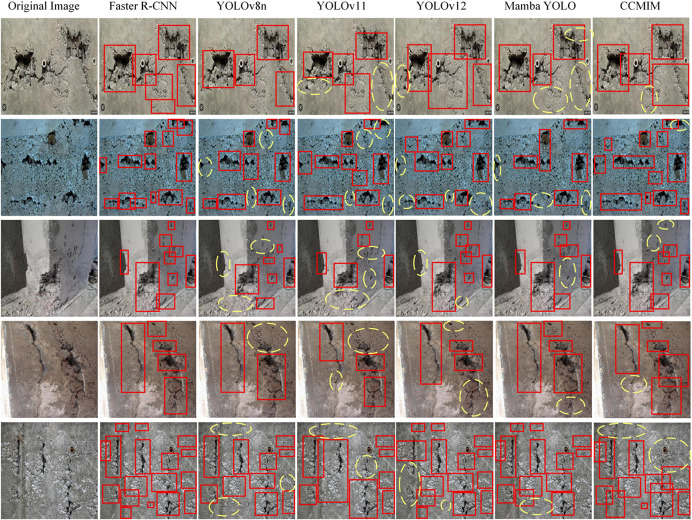
Qualitative comparison results of CCMIM on the CCCD dataset.

Figs 5, 6 and 7 show the qualitative analysis of the RDD2022, SDNET2018 and CCCD datasets, respectively. It is obvious from the figures that the proposed CCMIM method has a significant improvement over other algorithms in detecting fine cracks.

In the case of the RDD2022 dataset (Fig 5), traditional methods such as Faster R-CNN and YOLO series models usually have low confidence in detecting small cracks, and sometimes even miss small cracks, resulting in missed detections. These methods often misjudge background textures or noise as cracks, resulting in false detections. In contrast, CCMIM is able to detect small cracks with high confidence in complex background scenes, significantly reducing missed detections and false detections.

For the SDNET2018 dataset (Fig 6), the YOLOv8n and Faster R-CNN models show low confidence in dealing with fine cracks on concrete bridge decks, and sometimes fail to correctly identify small cracks, resulting in missed detections. On the other hand, CCMIM consistently detects small cracks with high confidence and performs well in complex backgrounds, effectively avoiding missed detections and reducing false detections.

In the case of the CCCD dataset (Fig 7), methods such as YOLOv11 and Deformable-DETR perform poorly in detecting small cracks in building structures, have low confidence, and occasionally misidentify background features as cracks, leading to false detections. In contrast, CCMIM shows stronger performance, is able to confidently identify cracks, and significantly reduces missed detections and false detections.

### 4.5 Model parameter analysis

The CCMIM model performs well in multiple indicators, especially in computational efficiency. Its parameter size is 2.88M, which is significantly lower than Deformable-DETR (42.3M) and Efficient-DETR (34.5M), significantly reducing the consumption of computing resources. The smaller parameter size reduces memory and computing requirements while maintaining high accuracy, making it suitable for deployment on resource-constrained devices. CCMIM’s GFLOPs is 6.3, much lower than Deformable-DETR (36.8 GFLOPs) and Efficient-DETR (28.9 GFLOPs), ensuring efficient reasoning.

**Fig 8:**
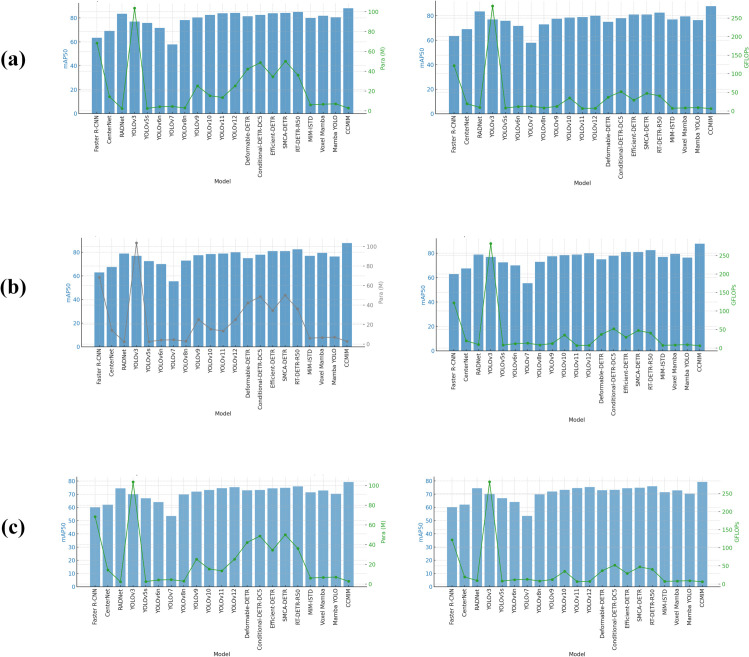
The relationship between model parameters, computational complexity, and mAP50 of CCMIM on the RDD2022, SDNET2018, and CCCD datasets. (a) RDD2022 dataset, (b) SDNET2018 dataset, (c) CCCD dataset.

Fig 8 shows the relationship between model parameters, computational complexity, and mAP50 on RDD2022, SDNET2018, and CCCD datasets. Although CCMIM has lower parameters and computational complexity, its mAP50 index is significantly higher than other methods, showing that it optimizes the number of parameters and computational complexity while improving accuracy, reducing computing resource consumption.

### 4.6 Ablation experiment

The results in Table 6 show that, first, after removing the MiM module, the F1 score and mAP50 of the model on the three datasets of RDD2022, SDNET2018 and CCCD dropped by an average of about 30%, indicating that the MiM module is crucial in capturing detailed features and recognizing objects in complex backgrounds. Secondly, after removing the SPT module, the F1 score and mAP50 dropped by an average of about 20%. The SPT module improves the performance of the model in complex backgrounds through multi-scale feature fusion and global context modeling. After removing the MHA module, the F1 score and mAP50 dropped by an average of about 10%-15%. The MHA module is indispensable in capturing long-range dependencies and global information. Finally, after removing the DDF module, the F1 score and mAP50 dropped by an average of about 10%. The DDF module plays an important role in the fusion of local and global features, especially enhancing the adaptability of the model through the ECA and DF mechanisms.

**Table 6:** Ablation study.

Method	Datasets					
	RDD2022		SDNET2018		CCCD	
	F1 Score	mAP50	F1 Score	mAP50	F1 Score	mAP50
Full CCMIM Model	85.8	88.1	82.3	87.8	78.2	79.2
w/o MiM Block	52.3	53.7	48.5	51.0	46.3	48.5
w/o SPT	58.7	61.0	55.6	57.1	53.1	54.6
w/o MHA	70.2	72.5	65.9	67.6	63.0	64.5
w/o DDF	66.3	68.7	62.2	64.0	59.1	60.5

Overall, these experimental results show that each module in the CCMIM model plays an irreplaceable role in accuracy and robustness. The MiM and SPT modules are particularly outstanding in detail capturing and multi-scale information fusion, while the MHA and DDF modules play important roles in enhancing global information modeling and feature fusion. Removing any module results in a significant performance decline, further proving the superiority of the model architecture.

**Effectiveness of the MiM Block Module.** As shown in Table 7, we conducted a second ablation study to evaluate the performance of different Mamba models and compare them with CCMIM. In this experiment, we replaced the MiM module in the CCMIM model with Vim-Ti, Vim-S, VSS module, and Famba-V.

**Table 7:** Ablation study.

Method	Datasets					
	RDD2022		SDNET2018		CCCD	
	F1 Score	mAP50	F1 Score	mAP50	F1 Score	mAP50
Full CCMIM Model (MiM Block)	85.8	88.1	82.3	87.8	78.2	79.2
Vim-Ti	79.6	81.4	77.0	81.2	73.8	75.2
Vim-S	80.7	82.1	78.0	81.6	74.5	75.9
VSS Module	81.2	83.0	79.3	82.5	75.1	76.4
Famba-V	80.0	82.2	79.8	82.0	74.2	75.1

From the experimental results, the Full CCMIM Model (MiM Block) outperforms other models across all datasets. Specifically, on the RDD2022 dataset, CCMIM achieved an F1 Score of 85.8 and an mAP50 of 88.1, significantly outperforming Vim-Ti (F1 Score 79.6, mAP50 81.4), Vim-S (F1 Score 80.7, mAP50 82.1), and other comparison methods. On both the SDNET2018 and CCCD datasets, CCMIM also demonstrated its superiority, with F1 Score and mAP50 improving by 2-5 percentage points, demonstrating the critical role of the MiM module in enhancing model accuracy. Notably, Vim-Ti and Vim-S performed reasonably well on some datasets, but their F1 Score and mAP50 were still significantly lower than CCMIM. The VSS module performed relatively better, especially on the RDD2022 dataset, where its mAP50 (83.0) showed some improvement compared to other methods, but it still lagged behind CCMIM. Famba-V showed performance closer to Vim-S, indicating that the CCMIM model, with its integration of the MiM module, has a significant advantage, particularly in fine detail capture, background processing, and small target recognition.

### 4.7 Limitations and future directions

Although the proposed CCMIM model demonstrates excellent performance in concrete defect detection tasks and provides an effective solution for defect recognition in cement-based materials, there are still three key limitations when considering practical engineering applications and technological advancements. First, the model’s noise robustness needs further improvement. In real-world concrete defect detection scenarios, the cement surface is often subject to noise such as stains, texture interference, and uneven lighting (e.g., strong reflections or local shadows). While the current model mitigates background interference through the ECA mechanism and dynamic filters in the DDF module, its ability to extract crack features significantly decreases in high-noise environments (e.g., cracks obscured by large areas of oil stains or blurred images under low light). Specifically, the detection rate of microcracks smaller than 0.2mm increases in terms of false negatives or false positives compared to noise-free scenarios, making it difficult to stably adapt to detection requirements in complex noisy environments. Second, there is room for improvement in modeling complex crack shapes and multi-scale features. The current model uses the MiM module’s “visual sentence - visual word" hierarchical parsing mechanism to integrate global and local features. However, it struggles to model common complex crack shapes in cement-based materials in civil engineering (e.g., mesh-crossing cracks, branching cracks) and cross-scale cracks (e.g., the simultaneous presence of 0.1mm microcracks and 2mm regular cracks within the same image). The crossing nodes of mesh cracks are easily misjudged as independent small cracks, and the weight distribution of cross-scale crack features lacks dynamic adjustment mechanisms, leading to a reduction in the model’s mAP50 for these complex cracks compared to simple crack shapes. This indicates that the model has not fully captured the diversity of crack shapes in real-world cement-based materials. Finally, although comprehensive validation has been conducted on three representative datasets—RDD2022, SDNET2018, and CCCD—covering typical scenarios like roads, bridges, and buildings, the overall datasets still have limitations in terms of regional distribution, crack types, and lighting conditions. The model’s generalization ability has not been fully validated in more complex real-world scenarios. Existing datasets primarily focus on defect samples of ordinary cement concrete in standard environments (normal temperature and dryness) and do not cover cracks caused by cement pore structure degradation in extreme environments (e.g., high humidity, freeze-thaw cycles). Additionally, there is a lack of crack data for high-performance cement-based composite materials (e.g., fiber-reinforced cement concrete), leading to a decline in the model’s detection accuracy in these unknown scenarios, and its generalization capability is insufficient to support multi-scenario and multi-material engineering applications.

To address these limitations, future work can proceed in three directions: First, enhancing noise robustness by introducing a noise-adaptive suppression submodule based on diffusion models, dynamically generating noise cleaning strategies in conjunction with cement pore structure texture features to reduce the impact of high-concentration interference on crack feature extraction, especially improving the stability of microcrack detection in complex noisy environments. Second, optimizing the modeling ability of complex cracks and multi-scale features by improving the hierarchical parsing logic of the MiM module, adding crack shape classification units (e.g., mesh and branching crack recognizers based on contour features), and designing a dynamic weight algorithm for cross-scale features to achieve precise feature matching for cracks of different shapes and scales. Third, improving the model’s generalization performance in unknown extreme conditions or for different materials by constructing a multi-source heterogeneous dataset covering extreme environments (e.g., freeze-thaw, high humidity) and special cement-based materials. This can be combined with transfer learning and domain adaptation training methods to enable the model to learn the crack feature patterns of cement-based materials under different conditions, ultimately expanding the application scope of the CCMIM model in the field of concrete structure health monitoring.

## 5 Conclusion

The CCMIM model introduced herein incorporates the MiM module, SPT module, and DDF module, demonstrating superior performance in the domain of concrete defect detection, particularly in the identification of minute cracks and complex environments. Empirical validation illustrates that the CCMIM model achieves significant advancements over conventional techniques and extant methodologies premised on YOLO, Transformer, and Mamba frameworks, specifically in terms of critical performance indicators such as detection accuracy, recall, F1-score, and mAP50 across various datasets, including RDD2022, SDNET2018, and CCCD. Notably, in the precise detection of small cracks, the CCMIM model proficiently mitigates both missed detections and false positives, thereby exhibiting enhanced robustness and precision. In summary, the CCMIM model demonstrates exceptional efficacy in detecting cracks in bridges, roads, and buildings, promising extensive practical applications. Prospective research endeavors could aim to further refine the model’s real-time processing capabilities and inference velocity, alongside investigating more effective multimodal feature fusion strategies, thereby furnishing more robust technical support for crack detection across diverse engineering disciplines.
